# Joint Association of Sitting Time and Physical Activity with Metabolic Risk Factors among Middle-Aged Malays in a Developing Country: A Cross-Sectional Study

**DOI:** 10.1371/journal.pone.0061723

**Published:** 2013-04-17

**Authors:** Anne H. Y. Chu, Foong Ming Moy

**Affiliations:** Julius Centre University of Malaya, Department of Social and Preventive Medicine, Faculty of Medicine, University of Malaya, Kuala Lumpur, Malaysia; Cardiff University, United Kingdom

## Abstract

**Background:**

Prolonged sitting is associated with increased weight and higher risks for abdominal obesity, dyslipidaemia, hyperglycaemia and hypertension among the adult population. This has been well documented in the West, but studies on these associations are lacking in developing countries, including Malaysia.

**Objective:**

This cross-sectional study aimed to examine the joint association of sitting time and physical activity with metabolic risk factors among middle-aged working adults.

**Methodology:**

A total of 686 Malay men and women participated (mean age 45.9±6.5 years). Metabolic syndrome was diagnosed from the modified NCEP ATP III criteria. Self-reported sitting time was obtained with the validated Malay version of the International Physical Activity Questionnaire. Participants were asked about their time spent sitting during travel in a motor vehicle, e.g., car, motorcycle or bus, over the preceding 7 days. Logistic regression was used to estimate the odds ratio with the confidence interval for the combined effects of sitting quartiles and physical activity categories with metabolic risk factors.

**Results/Significance:**

The prevalence of metabolic syndrome among our participants was 31.9%. Their average total sitting time (including transportation) was 7.6±2.4 h/day. After we adjusted for gender and educational level, higher sitting quartiles and physically inactive groups were associated with higher odds for metabolic syndrome compared with the referent group (sitting <6 h/day and physically active). In the physically active stratum, the odds for metabolic syndrome in participants who sat ≥9.3 h/day was 3.8 times that of participants who sat <6 h/day. Both higher sitting quartiles and insufficient physical activity were associated with adverse effects on abdominal obesity, hypertriglyceridemia and hyperglycaemia.

**Conclusion:**

In joint analyses of sitting time and physical activity, higher sitting time and insufficient physical activity were deleteriously associated with odds for metabolic risk factors in middle-aged Malay men and women.

## Introduction

In recent years, sedentary behaviour has become an emerging public health concern among the adult population. Sedentary behaviour includes activities such as sitting, television viewing, lying down and riding in a car [1], all of which require comparatively low levels of total energy expenditure in the range of 1.0–1.5 metabolic equivalents (METs) [2]. According to a recent survey on sitting behaviour from the International Physical Activity Questionnaire (IPAQ) in 20 countries, 49,493 adults self-reported a median of 5 h/day spent sitting, ranging from ≤3 h/day in Portugal, Brazil and Colombia to ≥6 h/day in Taiwan, Norway, Hong Kong, Saudi Arabia and Japan [3]. In the United States, time-use surveys have documented that adults who hold a full-time job spend much of their working hours sitting in an office and spend an average of over 2 h/day watching television or playing computer games [4]. A rapid rise in dependency on motorized road vehicles as the main mode of transportation has been witnessed in the capital city of Malaysia–Kuala Lumpur [5]. Increasing vehicle ownership leads to congestion on the road, resulting in longer travel time. The most adverse impact is experienced by working adults who spend large amounts of time commuting during rush hour.

Lack of physical activity is also known to be an established risk factor in the development of metabolic risk factors [6]. Metabolic syndrome refers to a constellation of metabolic and cardiovascular risk factors, including abdominal obesity, dyslipidaemia, hyperglycaemia and hypertension, which inevitably results in type-2 diabetes and cardiovascular diseases [2]. This endemic is no longer limited to developed countries: it has expanded to many low- to middle-income developing countries that have entered an ‘epidemiological transition’ [3]. Although a few studies have evaluated the joint association of sedentary behaviour and physical activity with health outcomes, they have been limited to body mass index (BMI) [7], [8] or have been conducted in Western countries [7], [9], [10]. By stratifying analysis according to physical activity level and gender, one study in the United States that investigated leisure-time sedentary behaviour and physical activity reported higher odds for metabolic syndrome [11].

As there has been increasing research in this important area, attention should also be paid to Asian counterparts. Sitting at work and during transport may be the most common sedentary behaviour that Malaysian adults engage in, like that of most working adults. Because residents of Kuala Lumpur are now faced with increased dangerous levels of obesity and chronic health problems associated with sedentary lifestyles and insufficient physical activity [12], we aimed to examine the joint association of sitting time (transportation included) and physical activity with metabolic risk factors among middle-aged Malay working adults.

## Methods

### Study design

This was an analytical cross-sectional study.

### Sample size calculation

Sample size was calculated using the Power and Sample Size (PS) Program. Referring to a local study on the comparison between different levels of physical activity with BMIs among men ranging in age from 18 to 44 years [12], with a significance level pre-set at 0.05, within group standard deviation equal to 3.5 and difference in group means of 0.9, the required minimum sample size was 476. On the basis of a previous pilot survey, the sample size was adjusted to accommodate an expected 50% of non-respondents; therefore, the adjusted sample size was 1000.

### Setting

The study was conducted between August 2010 and August 2011 in a public university in Kuala Lumpur, Malaysia.

### Eligibility

Employees who participated in the university's annual health screening program, aged 35 years and over, who self-identified as Malay and were able to read and write well enough to record past activities were invited to participate in our study. People with physical disabilities and pregnant women were excluded. Ethics clearance was obtained from the Medical Ethics Committee of the Faculty of Medicine, University of Malaya (Reference Number: MEC 782.18). Written informed consent was obtained from all participants prior to the study.

### Measurement of sitting time and physical activity

The self-reported, validated long form of the Malay version of IPAQ (IPAQ-M) was used to assess sitting time [13]. Participants completed the IPAQ-M, in which they were asked about their (1) overall time spent sitting and (2) time spent sitting during travel in a motor vehicle (e.g., car, motorcycle or bus) over the preceding 7 days. The overall time spent sitting was calculated by summing the time spent sitting on weekdays and weekends in the office or at home. The time spent sitting in a motor vehicle was assessed separately in the questionnaire, which asked about the time spent travelling back and forth regularly, such as between one's place of work and home. The total sitting time (including transportation) in this study was reported as hours per day (h/day) and calculated as follows: [(weekday sitting×5)+(weekend sitting×2)/7+time spent sitting in a motor vehicle]. From this calculation, we estimated the average time spent sitting per day. Continuous variables obtained from the total sitting time (including transportation) were further categorized into quartiles (<6 h/day, 6–7.59 h/day, 7.6–9.29 h/day, ≥9.3 h/day).

The levels of physical activity among the participants were also assessed with IPAQ-M. Participants were asked to recall the type and duration of their physical activities in the last 7 days. For the analysis of physical activity data, the following MET-values were used: walking = 3.3 METs, moderate physical activity = 4.0 METs and vigorous physical activity = 8.0 METs. The results were presented as the estimation of energy expenditure in metabolic equivalent-minutes per week (MET-min week^−1^). The MET-min week^−1^ was calculated as follows: minutes of activity/day×days per week×MET-value. From this continuous variable of total physical activity scores, the data were categorised according to the IPAQ scoring guidelines. Participants with a total physical activity of <600 MET-min week^−1^ were classified in the ‘low’ category, 600–2999 MET-min week^−1^ in the ‘moderate’ category, and ≥3000 MET-min week^−1^ in the ‘high’ category.

We previously validated the IPAQ-M among a Malay population in Malaysia [13]. The 7-day test-retest reliability of sitting time revealed a good intra-class correlation coefficient of 0.84 (95% confidence interval [CI]: 0.77–0.90, p<0.001), and validity against a 7-day physical activity log book was 0.86 (Spearman's ρ). The intra-class correlation coefficient for the assessment of physical activity revealed moderate to good reliability ranging from 0.54–0.92 (p<0.001), and a kappa coefficient of 0.89 showed good validity (95% CI: 0.79–0.98).

### Socio-demographic, anthropometric and body composition measurements

Data on demographic characteristics (age, gender, education level, occupational status), anthropometric parameters (weight, height and waist circumference), systolic/diastolic blood pressure, fasting blood glucose and fasting lipid profile were collected after participants were instructed to fast overnight (at least 8 h) prior to blood collection. Weight was measured using the SECA digital scale, height by the SECA stadiometer (Hamburg, Germany). BMI was calculated using the formula of weight (kg) divided by height^2^ (m^2^). From the international classification of adult underweight, overweight and obesity according to BMI [14], a BMI below 18.5 is considered to be underweight, a BMI of 18.5 to 24 healthy, a BMI of 25 to 29 overweight, and a BMI of 30 and above obese. Waist was measured in a standing position with a circumference measurement tape. The waist was taken as the point midway between the iliac crest and the costal margin (lower rib). All measurements were performed by trained staff and quality checks were conducted regularly. Blood pressure was measured using a clinically validated digital automatic blood pressure monitor (Omron HEM-907 model, Kyoto, Japan) after at least 10 minutes of seated rest. Two blood pressure readings were obtained and averaged for use in the analysis.

The analysis of biochemical indicators, which included fasting blood glucose and full lipid profile, were conducted by the Clinical Diagnostic Laboratory of the University Malaya Medical Centre. Fasting serum total cholesterol, triglyceride and plasma glucose levels were measured using an automated analyser (Siemens Healthcare Diagnostics Inc., Newark, NJ, USA). For the total cholesterol assayed, the intra-assay and inter-assay coefficients of variation were 0.84% and 1.3%, respectively, with a sensitivity of 1.3 mmol/L (50 mg/dL). For the triglycerides assayed, the intra-assay and inter-assay coefficients of variation were 0.4% and 1.3%, respectively, with a sensitivity of 0.17 mmol/L (15 mg/dL). For the glucose assayed, the intra-assay and inter-assay coefficients of variation were 0.6% and 1.6%, respectively, with a sensitivity of 0 mmol/dL (1 mg/dL). Fasting serum high-density lipoprotein (HDL) cholesterol levels were measured using an automated analyser (Dade Behring Inc., Newark, NJ, USA). The intra-assay and inter-assay coefficients of variation were 1.57% and 2.00%, respectively, with a sensitivity of 0.26 mmol/L (10 mg/dL) for the HDL cholesterol assayed. Low-density lipoprotein (LDL) cholesterol levels were calculated using the Friedewald formula as follows: LDL cholesterol (mmol/L) = total cholesterol (mmol/L)−HDL cholesterol (mmol/L)−[triglycerides (mmol/L)/2.2]. LDL cholesterol levels were not calculated if the sample triglyceride levels were ≥4.5 mmol/L because the Friedewald equation for the LDL cholesterol estimation was not calibrated.

### Definition of metabolic syndrome

From the modified NCEP ATP III criteria, which is more suitable for the Malay population [15], the presence of at least three of the following five factors is required for a working definition of metabolic syndrome: (1) abdominal obesity; (2) hypertriglyceridaemia (triglycerides ≥1.7 mmol/L) or specific treatment for this lipid abnormality; (3) low HDL cholesterol (HDL cholesterol ≤1.03 mmol/L for men and ≤1.29 mmol/L for women) or specific treatment for this lipid abnormality; (4) elevated blood pressure (systolic blood pressure ≥130 mmHg and/or diastolic blood pressure ≥85 mmHg) or current use of antihypertensive drugs; (5) impaired fasting glucose (fasting plasma glucose ≥5.6 mmol/L) or drug treatment for elevated glucose (previously diagnosed type 2 diabetes). The NCEP ATP III criteria suggested the cut-off points of waist circumference should be ethnic specific, with adoption of the Asia criteria for abdominal obesity (waist circumference >90 cm in men and >80 cm in women).

### Statistical analysis

Data were entered and analysed using SPSS statistical software for Windows version 16.0 (SPSS, Inc., Chicago, IL). All statistical tests were two-sided and the significance level was set at p<0.05. CIs were estimated at the 95% level.

The chi-squared test for categorical variables, independent *t*-tests for normally distributed continuous variables and Mann-Whitney tests for nonparametric variables were used to analyse the differences in characteristics between men and women. One-way analysis of variance (ANOVA) was used to examine differences in sitting time between categorical socio-demographic characteristics. Bonferroni's post-hoc test was used to perform pairwise comparisons between continuous sitting time variables and socio-demographic variables with more than two categories if significant differences were found in the ANOVA.

We investigated the interaction between sitting quartiles and physical activity categories for the odds of developing metabolic risk factors. In the analysis, the physical activity variable was dichotomized to two categories of meeting the physical activity recommendations versus not meeting the physical activity recommendations. These physical activity recommendations were based on the public health guidelines from the US Surgeon General [16]. The guidelines of 30 minutes of moderate-intensity activity 5 days a week, 20 minutes of vigorous activity 3 days a week, or an equivalent combination of moderate- and vigorous-intensity activity were equivalent to the ‘moderate’ category obtained from the IPAQ [17]. If the interaction term was statistically significant, joint sitting and physical activity variables would be created by dividing the participants into groups on the basis of their sitting quartiles and two different categorizations of physical activity. Logistic regression was used to estimate odds ratios (ORs) with CIs for the relation between sitting quartiles and metabolic risk factors in the strata of the physical activity levels. The lowest sitting quartile and category of meeting the recommended physical activity level was always the referent group. The potential confounders adjusted for were gender (men, women) and educational level (primary, secondary or tertiary).

## Results

A total of 686 Malay employees participated in this study (response rate of 68.6%). Socio-demographic, anthropometric and clinical characteristics of the study's participants are shown in Table 1. The study population consisted of 60.3% women. The mean BMI of this population was 27.2±4.8 kg/m^2^. Individuals who were identified as overweight and obese predominated (65.6%). More women than men were engaged in low physical activity levels (p<0.001) and had a higher prevalence of obesity (p<0.001). Participants with metabolic syndrome were more often men (p = 0.02). Participants spent an average of 6.4 h/day sitting. The sitting time increased approximately 1 to 3 h after combining it with transport-related sitting time.

**Table 1:** Characteristics of the participants.

	Total (*n* = 686)	Men (*n* = 272)	Women (*n* = 414)	p-value*
Characteristic	*n* (%)	*n* (%)	*n* (%)	
Age group				0.14
40 years and below	139 (20.3)	52 (19.1)	87 (21.0)	
41–49 years	349 (50.9)	131 (48.2)	218 (52.7)	
50 years and above	198 (28.8)	86 (31.6)	108 (26.1)	
Mean ± SD	45.9±6.5	46.4±6.9	45.5±6.0	
Educational level				0.01
Primary	142 (20.7)	66 (24.3)	76 (18.4)	
Secondary	247 (36.0)	80 (29.4)	167 (40.3)	
Tertiary	297 (43.3)	126 (46.3)	171 (41.3)	
Occupational status				0.03
Support group (unskilled)	181 (26.4)	81 (29.8)	100 (24.2)	
Support group (skilled)	288 (42.0)	109 (40.1)	179 (43.2)	
Professional (non-academic)	56 (8.2)	13 (4.8)	43 (10.4)	
Professional (academic)	161 (23.5)	69 (25.4)	92 (22.2)	
Levels of physical activity				<0.001
Low (<600 MET-min week^−1^)	186 (27.1)	59 (21.7)	127 (30.7)	
Moderate (600–2999 MET-min week^−1^)	316 (46.1)	116 (42.6)	200 (48.3)	
High (≥3000 MET-min week^−1^)	184 (26.8)	97 (35.7)	87 (21.0)	
Total sitting time (h/day)				
Sitting only, mean ± SD	6.4±2.4	5.7±3.7	6.6±2.9	<0.01
Sitting (including transport) Mean ± SD	7.6±2.4	7.3±2.5	7.9±2.3	<0.001
Body mass index (kg/m^2^)†				<0.001
Normal (18.5–24)	236 (34.4)	89 (32.7)	147 (35.5)	
Overweight (25–29)	269 (39.2)	131 (48.2)	138 (33.3)	
Obese (≥30)	181 (26.4)	52 (19.1)	129 (31.2)	
Metabolic syndrome	219 (31.9)	101 (37.1)	118 (24.2)	0.02
Abdominal obesity^a^	373 (54.4)	113 (41.5)	260 (62.8)	<0.001
Hypertriglyceridaemia^b^	259 (37.8)	150 (55.1)	109 (26.3)	<0.001
Low HDL cholesterol^c^	229 (33.4)	83 (30.5)	146 (35.3)	0.19
Hypertension^d^	362 (52.8)	176 (64.7)	186 (44.9)	<0.001
Hyperglycaemia^e^	133 (19.4)	64 (23.5)	69 (16.7)	0.03

* Significant difference between men and women.

† Classification according to the World Health Organization (1998).

HDL: high-density lipoprotein; MET: metabolic equivalent.

a Abdominal obesity was waist circumference >90 cm in men and >80 cm in women.

b Hypertriglyceridaemia was ≥1.7 mmol/L or with treatment.

c HDL cholesterol was ≤1.03 mmol/L for men and ≤1.29 mmol/L for women or with treatment.

d Hypertension was ≥130/85 mmHg or with treatment.

e Hyperglycaemia was ≥5.6 mmol/L or with treatment.

Total sitting time (including transport) and socio-demographic characteristics of participants are presented in Table 2. Participants with a primary educational level had significantly less sitting time than those who had higher educational levels, but no significant difference was found between sitting time and secondary or tertiary education levels. Total hours of sitting time were significantly associated with categories of physical activity recommendations, with individuals who failed to meet the recommendations spending more time sitting. In contrast, no significant difference was found between sitting time and age groups or occupational status.

**Table 2:** Comparisons of sitting time and socio-demographic characteristics.

Characteristic	Total sitting time (including transport), h/day	p-value
Age group		0.28
40 years and below	7.4±2.4	
41–49 years	7.7±2.4	
50 years and above	7.7±2.3	
Educational level		0.03
Primary	7.2±2.5^a^	
Secondary	7.9±2.3^b^	
Tertiary	7.6±2.4^b^	
Occupational status		0.3
Support group (unskilled)	7.4±2.4	
Support group (skilled)	7.6±2.3	
Professional (non-academic)	7.9±2.4	
Professional (academic)	7.8±2.5	
Physical activity recommendations^c^		<0.001
Not meeting	8.4±2.2	
Meeting	7.7±2.4	

Data presented as mean ± SD.

p-values are from one-way ANOVA.

a,b Mean values with different superscript letters differ significantly from one another according to the Bonferroni test.

c Physical activity recommendations are based on the public health guidelines from the US Surgeon General [16].

**Figure 1:**
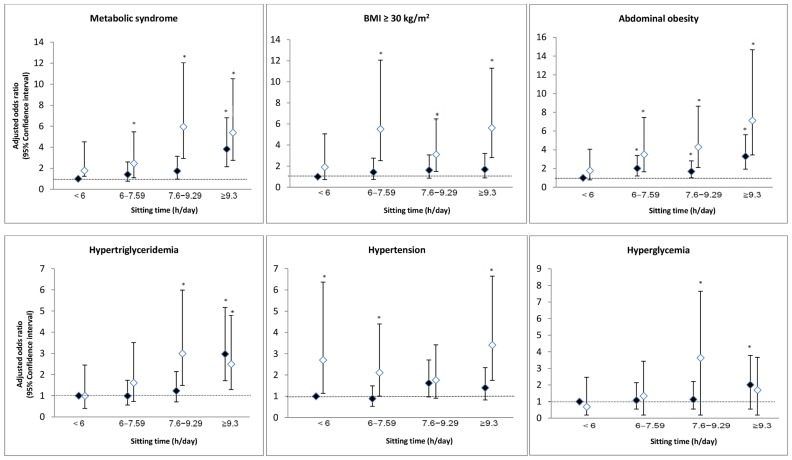
Joint association of sitting quartiles and physical activity levels for metabolic risk factors. Error bars indicate 95% CI. The referent group comprises participants with the lowest sitting quartile (<6 h/day) and being physically active. Analyses were adjusted for gender and educational level. *Significant difference (p<0.05) compared to referent group. Physical activity level ♦ Active ◊ Inactive.

Figure 1 depicts the joint associations of sitting time and physical activity with the odds for metabolic risk factors (p for interaction was <0.05 between sitting time and physical activity). Compared with the referent group of sitting <6 h/day and being physically active, both higher sitting quartiles and physically inactive groups were generally associated with increased odds for metabolic syndrome, after adjusting for gender and educational level. For example, even in the physically active stratum, the odds for metabolic syndrome increased 2.8-fold in participants who sat ≥9.3 h/day than for those who sat <6 h/day. Within the same physically inactive stratum, those categorized in the lower sitting quartile of 6–7.59 h/day had an OR of 2.46 (95% CI: 1.10–5.47); those in the higher sitting quartiles of 7.6–9.29 h/day and ≥9.3 h/day had an OR of 5.94 (95% CI: 2.93–12.05) and 5.38 (95% CI: 2.75–10.52), respectively. Therefore, increasing sitting time in the same category of physical inactivity was shown to increase the odds for metabolic syndrome. Within the highest sitting quartile of ≥9.3 h/day, the OR was higher in the physically inactive group than in the active group.

In similar joint analyses of sitting time and physical activity, higher ORs of 5.51 (95% CI: 2.52–12.06), 3.11 (95% CI: 1.50–6.48) and 5.63 (95% CI: 2.81–11.29) were reported among physically inactive individuals who sat 6–7.59 h/day, 7.6–9.29 h/day and ≥9.3 h/day, respectively. Those who sat ≥6 h/day across both strata of physical activity had significantly increased odds of developing abdominal obesity ranging from 1.69 to 7.12, with the exception of the group reporting <6 h/day of sitting and being physically inactive. Likewise, compared with the participants with the lowest sitting quartile who had sufficient levels of physical activity, those with higher sitting quartiles within both physical activity categories were associated with increased odds for hypertriglyceridaemia and hyperglycaemia. Only hypertension showed significant associations within the physically inactive group among higher sitting quartiles. By contrast, sitting time and physical activity showed no significant association with the odds for having low HDL cholesterol levels (Figure not shown).

## Discussion

The present study, conducted among a sample of the urban Malay middle-aged population living in Malaysia, showed detrimental associations between sitting time and metabolic risk factors. The prevalence of metabolic syndrome has continued to increase in Malaysia and is higher than in other Asian countries, with a significant increase by age, especially in the urban areas [18]. Our analyses showed interactions between sitting time and physical activity, identifying a positive association with metabolic syndrome and inconsistent patterns with metabolic risk factors. The odds of developing metabolic syndrome and other individual components of metabolic syndrome, with the exception of low HDL cholesterol levels, were generally higher among those who spent more than 6 h/day sitting. To our knowledge, this is the first study in Malaysia to report the combined associations of time spent sitting and physical activity with the odds of developing metabolic risk factors.

The results indicate an association between individuals who reported sitting at least 6 h/day and increased odds of developing metabolic syndrome. Compared with individuals who met current guidelines for physical activity, those who were insufficiently active had unfavourable outcomes within sitting quartiles. Even among those who met the recommended amount of physical activity, sitting more than 9.3 h/day was found to be significantly associated with 3.82-fold increased odds for metabolic syndrome. In other words, for individuals who spent large amounts of daily time sitting, physical activity did not seem to be protective against the odds of developing metabolic syndrome. This finding was supported by a systematic review and meta-analysis published by Edwardson et al. [19] who reported that people spending more time in sedentary behaviour (sitting) had 73% increased odds of developing metabolic syndrome, independent of physical activity and gender (OR = 1.73, 95% CI: 1.55–1.94). These outcomes indicated that prolonged overall sitting time could be an important determinant of metabolic syndrome even though an individual was sufficiently active according to the physical activity guidelines. It has been proposed that in response to the negative energy balance induced by physical activity, some active individuals compensate for the energy deficit by increasing sedentary periods in other parts of their day, or subsequently increasing food intake in response to hunger [10]. In addition, prolonged sitting largely diminishes the time for engagement in physical activity that can lead to reduction of total energy expenditure [20]. On the other hand, not all previous studies presented such a significant relationship after adjusting for physical activity [21], [22]. These contradictory results showing a non-significant relationship between sitting time and metabolic syndrome probably resulted from implementation of different cut-off values for categorization of total sitting time per day; hence, the data and interpretations of the studies may vary.

The investigation of the association between sitting time and physical activity with individual metabolic risk factors has produced mixed results. Positive results in the present study are consistent with those of other studies that showed significant independent associations of sedentary time with abdominal obesity [23], triglycerides [20], [23] and fasting glucose concentration [24], irrespective of physical activity. One plausible physiological mechanism responsible for the adverse effect of sitting time is the loss of local contractile activity from prolonged sitting, resulting in the deleterious suppression of lipoprotein lipase activity in skeletal muscle (necessary for HDL cholesterol production and triglyceride uptake) [25], [26]. Higher occupational sitting time may have accounted for these adverse effects among our cohort of working middle-aged adults. For those who spent most of their time working in front of the computer or caught in a traffic jam while commuting to and from work, prolonged sitting is inevitable, and it is negatively associated with the amount of physical activity. However, as we did not distinguish sitting during transport with other sitting domains, we cannot determine whether it is the most important contributing factor leading to metabolic syndrome. Further study investigating the distinctive domains in which sitting occurs, such as during television viewing or other recreational screen time in domestic environments, during meetings or computer use in occupational environments, and while travelling in automobiles or other transportation environments will be beneficial in understanding the correlates of sitting time and health outcomes [27].

Relatively higher odds for obesity and hypertension were seen in our study among those who failed to meet physical activity recommendations. The joint association between sitting time and physical activity with obesity was similar to that seen in a previous cross-sectional study of 10,984 US adults, which also found negative associations between sedentary behaviour and lower BMI among those with leisure-time activity [7]. This suggests that the odds for obesity may be associated with activity levels or engagement of sitting time (or both). As for those who did not meet physical activity recommendations in the present study, participants categorized in the third sitting quartile of 7.6 to 9.29 h/day were associated with the lowest odds for obesity compared with the other quartiles, presenting a U-shaped association. It is unclear why this was the case, but one possible explanation for this association could be unhealthy dietary behaviour. In our study, however, we did not collect information about this behaviour. Therefore, measurement of dietary intake and its effects on BMI need to be investigated in future studies.

Physical activity also favourably influences blood pressure, as reported in previous studies [28], [29]. This observation of the relationship between sitting behaviour and hypertension has also been seen in American men and women [30]. The investigators reported that the prevalence of elevated blood pressure among their employed adult population increased significantly with commuting distance. In theory, they suggested that a longer commuting distance, which is directly related to a longer time spent sitting, contributed to chronic stress as a result of traffic congestion. This stress was correlated to physiologic consequences such as high blood pressure. Furthermore, longer sitting time spent in commuting has become a habitual source of sedentary behaviour. These findings suggest that decreasing body weight and improving blood pressure should be encouraged in conjunction with a reduction in overall energy expenditure, highlighting the potential importance of physical activity—not just sedentary behaviour—as an independent factor.

Our findings on the association between sitting time and low HDL cholesterol levels were insignificant. This lack of a significant association in the current study was probably because our participants were middle-aged adults, and the selection criteria may have introduced a certain degree of bias towards the population who are at higher risk for low HDL cholesterol levels. Confirmation in prospective cohort studies may be needed in which different populations, methodology and statistical analysis used among different studies is taken into account.

The current study is limited by its cross-sectional study design, which makes it difficult to establish causality or specify the direction of causation between the metabolic risk factors and sitting time. For instance, it is possible that participants with metabolic risk factors or a higher BMI may have the tendency to sit longer and engage more extensively in other sedentary behaviours. Nevertheless, it is also unclear whether participants with longer sitting time subsequently increased their odds of developing metabolic syndrome. Future studies examining the longitudinal effect of prolonged sitting on the risk of developing metabolic risk factors would be instructive. Recall bias may also have been present because of the self-reported measure of sitting time. Future research should aim to prospectively collect data on sitting behaviour and physical activity with an objective measure, such as the accelerometer. In addition, our study did not include diet as a potential confounding factor, which may influence the fasting lipid, blood pressure and glucose levels among adults. Moreover, the findings have limited generalizability, as our study population consists of middle-aged Malays from the urban setting who are well educated. Because most participants were office workers with consistent working hours and sitting patterns, this would bias the findings towards a study population that has a comparatively higher risk of developing metabolic risk factors.

Despite these limitations, our study has several strengths. Unlike other studies that have mainly focused on reporting leisure-time sedentary behaviour (television viewing), the sitting time in our study incorporated time spent commuting to and from work. In addition, the tool that we used to assess sitting time and physical activity was the IPAQ, which has been shown to have high reliability and validity. To better understand the link between sitting time and metabolic syndrome, experimental studies need to be conducted on the dose-response relationships and also the possible causal relationships. Large randomised controlled trials assessing the underlying interactions between physical activities and sitting time could provide more definitive evidence.

In conclusion, this study shows that combined effects of sitting time and physical activity were positively associated with metabolic syndrome in middle-aged Malay adults. The adverse effects of prolonged sitting on the odds of metabolic risk factors—particularly abdominal obesity—were also found across physical activity categories. For those who did not meet physical activity recommendations, prolonged sitting was associated with higher odds for obesity and hypertension. Our findings suggest that to decrease the odds of individuals developing metabolic risk factors, it is crucial to reduce the amount of time spent sitting in conjunction with promotion of physical activity. Future public health programs and interventions may need to be encouraged to focus on other sedentary behaviours and physical activity in the prevention of metabolic risk factors.
